# Examining the Link between Patient Satisfaction and Adherence to HIV Care: A Structural Equation Model

**DOI:** 10.1371/journal.pone.0054729

**Published:** 2013-01-30

**Authors:** Bich N. Dang, Robert A. Westbrook, William C. Black, Maria C. Rodriguez-Barradas, Thomas P. Giordano

**Affiliations:** 1 Houston Veterans Affairs Health Services Research and Development Center of Excellence, Houston, Texas, United States of America; 2 Michael E. DeBakey Veterans Affairs Medical Center, Houston, Texas, United States of America; 3 Department of Medicine, Baylor College of Medicine, Houston, Texas, United States of America; 4 Harris Health System, Houston, Texas, United States of America; 5 Jesse H. Jones Graduate School of Business, Rice University, Houston, Texas, United States of America; 6 E. J. Ourso College of Business, Louisiana State University, Baton Rouge, Louisiana, United States of America; University of Ottawa, Canada

## Abstract

**Introduction:**

Analogous to the business model of customer satisfaction and retention, patient satisfaction could serve as an innovative, patient-centered focus for increasing retention in HIV care and adherence to HAART, and ultimately HIV suppression.

**Objective:**

To test, through structural equation modeling (SEM), a model of HIV suppression in which patient satisfaction influences HIV suppression indirectly through retention in HIV care and adherence to HAART.

**Methods:**

We conducted a cross-sectional study of adults receiving HIV care at two clinics in Texas. Patient satisfaction was based on two validated items, one adapted from the Consumer Assessment of Healthcare Providers and Systems survey (“Would you recommend this clinic to other patients with HIV?) and one adapted from the Delighted-Terrible Scale, (“Overall, how do you feel about the care you got at this clinic in the last 12 months?”). A validated, single-item question measured adherence to HAART over the past 4 weeks. Retention in HIV care was based on visit constancy in the year prior to the survey. HIV suppression was defined as plasma HIV RNA <48 copies/mL at the time of the survey. We used SEM to test hypothesized relationships.

**Results:**

The analyses included 489 patients (94% of eligible patients). The patient satisfaction score had a mean of 8.5 (median 9.2) on a 0- to 10- point scale. A total of 46% reported “excellent” adherence, 76% had adequate retention, and 70% had HIV suppression. In SEM analyses, patient satisfaction with care influences retention in HIV care and adherence to HAART, which in turn serve as key determinants of HIV suppression (all p<.0001).

**Conclusions:**

Patient satisfaction may have direct effects on retention in HIV care and adherence to HAART. Interventions to improve the care experience, without necessarily targeting objective clinical performance measures, could serve as an innovative method for optimizing HIV outcomes.

## Introduction

Over 1.1 million people in the United States (US) live with HIV infection [1]. Poor retention in HIV care and suboptimal adherence to highly active antiretroviral therapy (HAART) remain major barriers to maximizing the benefit of effective treatment. Only about 60% of patients who know their HIV status get regular care [2]. Furthermore, among North American patients who access care and receive HAART, only about 55% take their medicines as prescribed [3]. Subsequently, despite the wide availability of effective treatment in the US, only approximately 1 in 4 patients with HIV infection achieve suppression of HIV replication [4]. Suboptimal HIV suppression carries serious individual and public health consequences, including the emergence of drug resistance, increased HIV-related complications, increased infectivity and secondary transmission, and worse survival [5], [6]. Thus, there is an urgent need to optimize HIV outcomes with interventions to retain patients in HIV care and promote adherence to HAART.

The business world offers a framework for increasing retention by focusing on customer satisfaction. Marketing studies clearly show that high satisfaction levels have a positive impact on customer loyalty, repeat patronage, and more extensive and favorable referrals [7]. Firms that appreciate this relationship view customer satisfaction as a useful metric for mapping customer retention strategies.

Analogous to the business model of customer satisfaction and retention, patient satisfaction could serve as an innovative focus for increasing retention in HIV care and adherence to HAART. Suppression of HIV replication represents the most important prognostic indicator for long-term survival with HIV infection. We sought to understand if patient satisfaction is related to suppression of HIV replication through its effects on retention in HIV care and adherence to HAART. We hypothesize that patient satisfaction positively impacts retention in HIV care and adherence to HAART, which in turn impact HIV suppression.

## Methods

### Study population

We used data from a cross-sectional study of patients receiving outpatient HIV primary care at Thomas Street Health Center (TSHC) and the Michael E. DeBakey Veterans Affairs Medical Center (VAMC) in Houston, Texas. This study took place within the context of a primary study to identify the drivers of overall satisfaction in patients receiving HIV primary care. A full description of the study design is described elsewhere [8]. The study detailed here was planned prior to primary data collection and represents the second phase of analysis. From January 13 to April 21, 2011, study staff screened all patients with a scheduled HIV primary care visit to preliminarily determine study eligibility. Eligibility requirements included: 1) age ≥18 years old; 2) time enrolled in clinic ≥1 year; and 3) having at least one HIV primary care visit in the past year. Patients incarcerated >30 days in the past year or who could not complete the survey due to mental, physical or language barriers were excluded from the study. Clinic exposure requirements ensured sufficient experience at the clinic to assess satisfaction over a 12-month time frame.

Due to limited study staff, we could not recruit all eligible patients concurrently. As such, we decided a priori to systematically sample patients from a list of eligible patients who had arrived at the clinic and checked in. Patients with the most recent check-in time at the time of study staff availability were approached for enrollment. The survey, available in English and Spanish, was administered prior to the HIV provider visit and took about 10 minutes to complete.

### Measures

#### Overall patient satisfaction

The survey instrument included 2 questions about overall care received in the clinic 1) “Overall, how do you feel about the care you got at this clinic in the past 12 months?” and 2) “Would you recommend this clinic to other patients with HIV?” These questions were adapted from validated patient self-report survey instruments [9], [10]. We converted responses for each question to a 0- to 10-point scale. Overall patient satisfaction was measured by averaging the response values of these 2 questions. Construct reliability was evaluated by calculating composite reliability and average variance extracted. We used recommended thresholds of 0.70 and 0.50, respectively [11]–[14].

#### Medication adherence

A validated, single-item measure assessed adherence to HAART. The item stated, “Many patients find it hard to take HIV medicines as their doctor prescribes them. In the past 4 weeks, how would you rate your ability to take all your HIV medicines as your doctor prescribed?” The 6-point response scale ranged from “very poor” to “excellent” [15], [16]. This item has been validated against medication event monitoring system data, an objective measure of adherence [15], and has an estimated reliability of 0.67 (personal communication, Y. Lee, 2012).

#### Retention in care

Since 2011, the US Department of Health and Human Services has recognized that patients with HIV suppression and a CD4 cell count well above the threshold for risk of opportunistic infection may need less intensive monitoring (e.g. clinicians may extend the interval for HIV RNA monitoring to every 6 months) [17]. Our definition of adequate versus inadequate retention in HIV care reflects clinical practice, where patients with stable clinical and immunological status can have follow-up intervals of 6 months (as opposed to the traditional 3–4 months). Retention in care was based on 1) the number of 3-month quarters with at least 1 completed HIV primary care visit in the year prior to survey completion (i.e. visit constancy) [18], and 2) HIV RNA and CD4 cell count results 1 year prior to survey completion ±60 days. Because some participants may be seen at imprecise intervals, and the last interval was bounded by the enrollment date, we extended the first quarter interval by 2 weeks on the front end. Patients with adequate retention in care had 1) 3 or 4 quarters with an HIV primary care provider visit, or 2) at least 2 quarters with an HIV primary care provider visit and HIV suppression 1 year prior to survey completion, or 3) at least 2 quarters with an HIV primary care provider visit, and both a CD4 cell count ≥500 and not yet prescribed HAART 1 year prior to survey completion. Patients not meeting these criteria were classified as having inadequate retention in HIV care.

#### HIV suppression

HIV suppression was defined as a plasma HIV RNA <48 copies/mL±30 days from the date of survey completion. Lab values were obtained from electronic medical records.

#### Other measures

Participants self-reported their gender, race, ethnicity, education, income, health status and incarceration history. The health status item was based on a validated, widely used question, “In general, how would you rate your overall health?” [10], [19]–[21]. The 5-point response scale ranged from “poor” to “excellent.” Validated, single-item questions identified participants with possible depression, excessive alcohol use, and illegal or prescription drug abuse [22]–[24]. Electronic medical and administrative records provided data on age, appointments and laboratory values (CD4 cell count and HIV RNA).

### Statistical Analysis

#### Relationship between Patient Satisfaction and Adherence to HIV Care

We compared overall patient satisfaction scores between participants with adequate versus inadequate retention in HIV care, “excellent” versus non-“excellent” adherence to HAART, and suppressed versus unsuppressed HIV replication using the Wilcoxon Rank-Sum Test.

#### Bivariate analyses

We performed bivariate analyses between potential control variables (demographic, health status, behavioral characteristics, and clinic utilization variables listed in Table 1) and all dependent variables in the structural equation model (patient satisfaction, retention in HIV care, adherence to HAART and HIV suppression). To be parsimonious in selecting control variables, we included only variables achieving a significance level of p<0.10 in bivariate analyses with at least 2 of the 4 dependent variables.

**Table 1:** Baseline characteristics of participants (N = 489).

Characteristics	
Age, years – mean (±SD)	48 (±11)
Gender – (%)	
Male	71
Female	29
Race ethnicity – (%)	
Non-Hispanic black	61
Non-Hispanic white	15
Hispanic	21
Other	3
Survey mode – (%)	
Self-administered	85
Interviewer-administered	15
Education – (%)	
Some high school or less	22
High school graduate or equivalent	35
Some college of higher	43
Household income – (%)	
≤$10K	54
>$10K and ≤$30K	36
>$30K	10
Depression screen, positive – (%)	43
Alcohol screen, positive – (%)	42
Illegal or Rx drug abuse screen, positive – (%)	19
Health status – (%)	
Poor/fair	20
Good/very good	65
Excellent	15
HIV risk factor – (%)	
IVDA	16
MSM, no IVDA	33
Heterosexual sex, no IVDA	50
Transfusion	<1
Currently prescribed HAART – (%)	94
Duration enrolled in clinic, years – mean (±SD)	7.6 (±4.5)
CD4 count^a^ – median (25^th^, 75^th^ percentiles)	449 (276, 665)

SD indicates standard deviation; IVDA intravenous drug abuse; MSM, men who have sex with men.

a Value closest to date of survey completion, ±30 days; CD4 cell count available for 85% of participants.

#### Structural equation modeling

We used structural equation modeling (SEM) to examine hypothesized relationships between patient satisfaction, retention in HIV care, adherence to HAART, and HIV suppression. SEM is a multivariate statistical method that: 1) inputs empirical data and qualitative causal assumptions from theory-based models, 2) allows for the simultaneous evaluation of direct, indirect and total effects of multiple variables, and 3) accounts for measurement error in the process of modeling relationships between latent variables (i.e. variables that are not directly observed, but estimated from directly measured ones).

Spearman's partial correlation coefficients were calculated for all measures in the structural modeling by controlling for age, race, ethnicity, depression and health status. These computations parcel out the shared variance between each control variable and pair of measures. The resulting partial correlation matrix was used as the input for the structural model estimation (Table 2). Missing data were treated by pairwise deletion. The correlations between clinic sites were comparable.

**Table 2:** Correlation Matrix.^a^

		1	2	3	4	5
1	Likelihood of recommending clinic	1.00				
2	Feelings about care	0.53**	1.00			
3	Adherence to HAART	0.11*	0.17**	1.00		
4	Retention in HIV care	0.17**	0.08	0.12*	1.00	
5	HIV suppression	0.11*	0.09	0.26**	0.26**	1.00

a Partial correlations controlling for age, race ethnicity, depression, and health status.

* p<0.05;

** p<0.01.

We first assessed the relationship between retention in HIV care, adherence to HAART and HIV suppression, controlling for age, race, ethnicity, depression and health status. This constituted the baseline model. Next, we included overall patient satisfaction as a predictor latent variable to determine its effect on the relationship between retention, adherence, and, ultimately, HIV suppression. We tested the hypothesized models using SPSS AMOS 19.0 statistical software (*SPSS* Inc, Chicago, IL).

We performed hypothesis testing by examining parameter estimates. The retention in HIV care and HIV suppression constructs were measured with single indicators. Since HIV RNA copies is the accepted standard measure of HIV suppression, the measurement loading for HIV suppression was set to 1.00 (i.e. no measurement error). Since no studies of reliability have been reported for the retention in HIV care construct and the construct is measured objectively, its measurement error was assumed to be 0 and the measurement loading was set to 1.00. The adherence to HAART construct has an estimated reliability of 0.67 (personal communication, Y. Lee, 2012). This was incorporated into the model by setting the measurement loading to 0.82 (the square root of the reliability 0.67) and the measurement error to 0.33 (1 minus the reliability 0.67).

Model goodness-of-fit was evaluated using 3 widely used indexes: chi-square test (χ^2^), the Comparative Fit Index (CFI) and Root Mean Square Error of Approximation (RMSEA) [14]. We used conventional cutoff criteria for fit indexes: 1) non-significant χ^2^ values, 2) CFI values >0.90 [25] or >0.95 [26], and 3) RMSEA values <0.06 [26] or <0.08 [27].

The Institutional Review Board (IRB) for Baylor College of Medicine and Affiliated Institutions approved this study. The IRB waived the need for written informed consent because this research involves no more than minimal risk to the participants. We collected verbal informed consent and documented the procedure. All data were de-identified and analyzed anonymously.

## Results

### Study population

The study sample includes 489 patients (94% of eligible patients approached; 388 from TSHC and 101 from VAMC). As shown in Table 1, the mean age was 48 years, 71% were men, 61% were non-Hispanic black, and 54% had a household income of ≤$10,000. Participants and eligible non-participants did not differ significantly in terms of age, race, sex, and ethnicity (data not shown).

### Overall patient satisfaction

Patients reported high levels of overall satisfaction with HIV care (mean = 8.5, SD = 1.7, median 9.2, range 0.8–10.0). Over 90% would “probably” (23.4%) or “definitely” (69.8%) “recommend this clinic to other patients with HIV,” and over 80% felt “mostly satisfied” (26.7%) or “completely satisfied” (57.3%) with their HIV care.

### Retention in HIV care

In the year before enrollment, 76% of participants had adequate retention in HIV care and 24% had inadequate retention. Participants with adequate retention were significantly more satisfied with their HIV care than patients with inadequate retention (median patient satisfaction score 9.17 versus 8.47, respectively; p = 0.02).

### Adherence to HAART

A total of 94% were “taking or supposed to be taking HIV medicines.” Among those prescribed HAART, 46%, 28%, 16%, 6%, 2% and 2% reported “excellent,” “very good,” “good,” “fair,” “poor,” and “very poor” adherence, respectively. Participants who reported “excellent” adherence were significantly more satisfied with their HIV care than patients who did not (median patient satisfaction score 10.00 versus 8.61, respectively; p<.0001).

### HIV suppression

HIV RNA values at the time of survey completion ±30 days were available for 84% of participants (N = 409). Seventy percent of these patients achieved HIV suppression. Participants who achieved HIV suppression were significantly more satisfied with their HIV care than patients who did not (median patient satisfaction score 9.17 versus 8.47, respectively; p<.01).

### Baseline model

**Figure 1:**
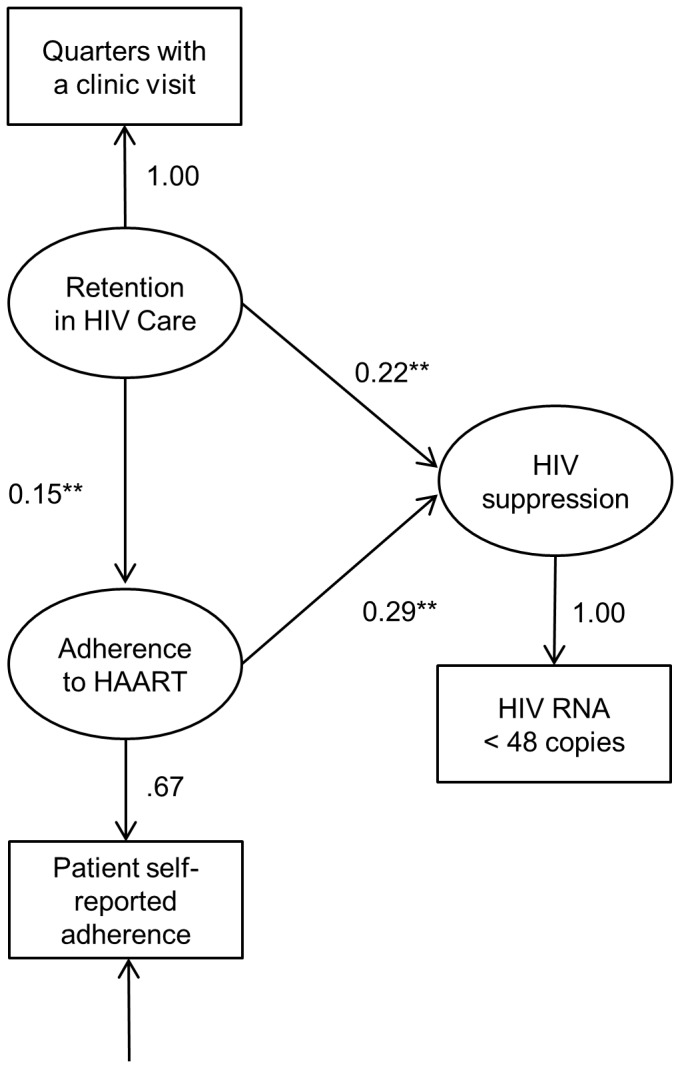
Baseline Model of Retention in HIV Care, Adherence to HAART and HIV Suppression (N = 489). Values indicate standardized coefficients; * p<0.05; ** p<0.001.

The baseline model evaluated the roles of retention in HIV care and adherence to HAART as independent antecedents to HIV suppression (Figure 1). The hypothesized model was a just-identified model with zero degrees of freedom. As such, the model did not allow a test of goodness-of-fit, since technically, all goodness-of-fit indexes in the estimated model have maximum values (χ^2^ = 0.00, *df* = 0, p = 0.00, CFI = 1.00, RMSEA = 0.00). However, the model still provides suitable estimates of the hypothesized relationships between latent variables. Table 3 shows the parameter estimates from the baseline model. Retention in HIV care and adherence to HAART were significantly associated with greater HIV suppression (standardized coefficient = .220, p<.0001 and standardized coefficient = .287, p<.0001, respectively).

**Table 3:** Parameter Estimates.

	B^a^	β	p
**Baseline Model** b			
Structural Model			
Retention in Care→Adherence to HAART	.147 (.062)	.147	.02
Retention in Care→HIV Suppression	.220 (.049)	.220	<.001
Adherence to HAART→HIV Suppression	.287 (.061)	.287	<.001
**Patient Satisfaction Model** c			
Measurement Model			
Patient Satisfaction→Feelings about care	1.000	.680	NA^d^
Patient Satisfaction→Recommend Clinic	1.149	.778	<.001
Structural Model			
Patient Satisfaction→Retention in Care	.266 (.094)	.181	<.001
Patient Satisfaction→Adherence to HAART	.298 (.115)	.203	<.001
Patient Satisfaction→HIV Suppression	.047 (.089)	.032	.60
Retention in Care→Adherence to HAART	.110 (.063)	.110	.08
Retention in Care→HIV Suppression	.215 (.050)	.215	<.001
Adherence to HAART→HIV Suppression	.280 (.062)	.280	<.001

B denotes B coefficient; β indicates beta coefficient.

Patient Satisfaction properties: composite reliability = 0.70; average variance extracted = 0.54.

a Standard errors in parentheses.

b Model Goodness of Fit: χ^2^ = 0.00, *df* = 0, p = 0.00, CFI = 1.00, RMSEA = 0.00.

c Model Goodness of Fit: χ^2^ = 5.106, *df* = 2, p = 0.078, CFI = 0.984, RMSEA = 0.064.

d NA indicates not applicable. The indicator loading is constrained to 1.0 for latent construct estimation and represents the reference item. No direct test of statistical significance is possible for the constrained indicator.

### Model of the effects of patient satisfaction

**Figure 2:**
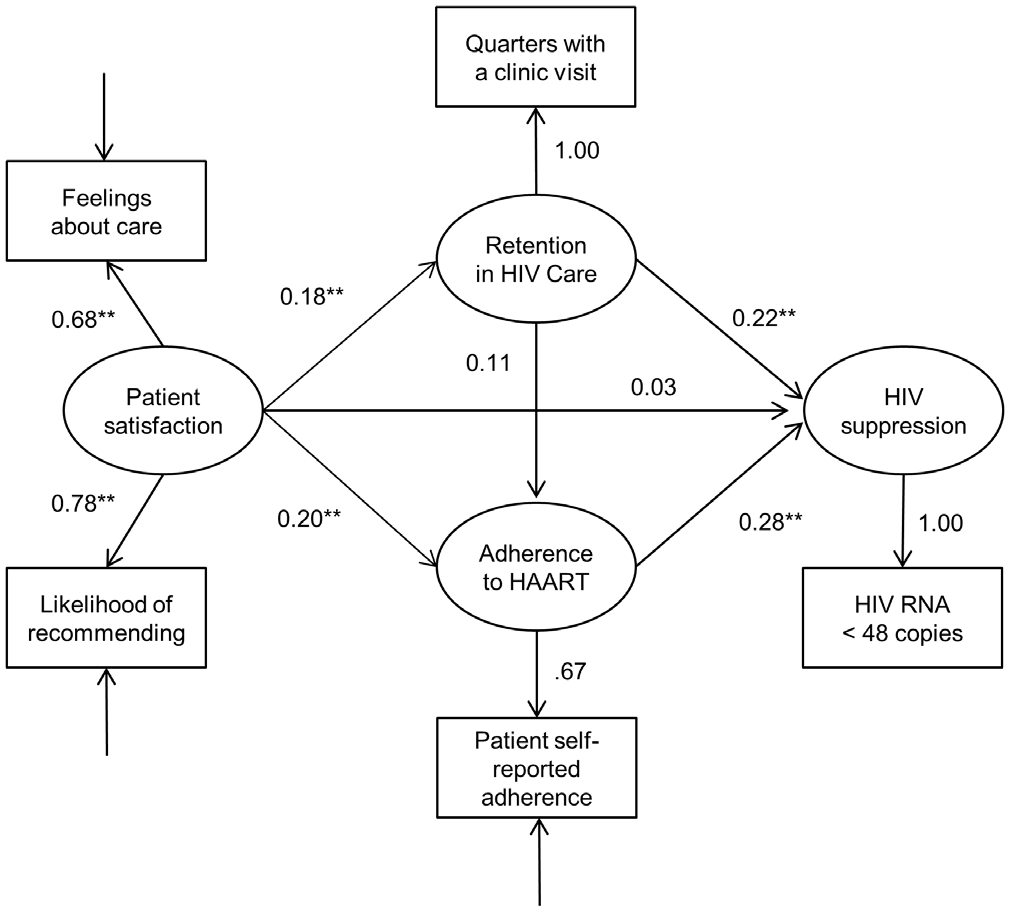
Patient Satisfaction Model (N = 489). Values indicate standardized coefficients; * p<0.05; ** p<0.001. Estimation requires that one of the indicator loadings of a construct be constrained to 1.0. No direct test of statistical significance is possible for this reference item. Statistical significance is determined by estimating an identical second model, with the indicator constraint of 1.0 moved to a different indicator. Thus, all standardized coefficients can be tested for significance, even though one item must always be constrained in any single estimation.

A second model evaluated the role of overall patient satisfaction in influencing retention in HIV care, adherence to HAART and HIV suppression (Figure 2). The hypothesized model provided a good fit to the data (χ^2^ = 5.11, *df* = 2, p = 0.08, CFI = 0.98, RMSEA = 0.06). Table 3 shows the parameter estimates from this hypothesized model of patient satisfaction. The composite reliability and average variance extracted tests for overall patient satisfaction exceeded recommended thresholds (values 0.70 and 0.54, respectively), indicating acceptable construct reliability [11]–[14].

Similar to the baseline model, the direct effects of retention in HIV care and adherence to HAART on HIV suppression were significant (standardized coefficient = 0.215, p<.0001 and standardized coefficient = 0.280, p<.0001, respectively) (Table 3). The direct effects of patient satisfaction on retention in HIV care and adherence to HAART were also significant (standardized coefficient = 0.181, <.0001 and standardized coefficient = 0.203, p<.0001, respectively). The direct effect of patient satisfaction on HIV suppression was not significant (standardized coefficient = .032, p = .60).

## Discussion

In this study of 489 participants receiving outpatient HIV primary care, overall patient satisfaction with care is positively related to retention in HIV care and adherence to HAART, which in turn serve as key determinants of HIV suppression. The data suggest that patient satisfaction may provide a way to improve HIV outcomes through its positive influences on adherence to HAART and retention in HIV care. This finding suggests that patient-centered interventions designed to improve the care experience could serve as an innovative method for optimizing HIV outcomes.

The National Council on Patient Information and Education's report, *Enhancing Prescription Medicine Adherence: A National Action Plan*, states that medication nonadherence has reached crisis proportions [28]. The report calls for adherence research that explores innovative ways to increase patient uptake of proven therapies. Successful interventions not only need to demonstrate efficacy and effectiveness, but also the capacity for ultimate adoption, implementation and maintenance in real-world settings.

Retention in HIV care is a critical step for achieving long-term survival with HIV infection [29]. Furthermore, HIV primary care guidelines recognize the importance of retention in HIV care as a precursor to adherence to HAART [30]. Successful strategies to improve retention in HIV care and adherence to HAART require an understanding of retention and adherence behavior and the complex interplay between biological, psychological, behavioral, social and health systems drivers. They also require a multi-level, multi-component approach to responding to the needs and concerns of individual patients. Simple practices shown to improve adherence include reductions in dose frequency and the use of adherence aids (e.g. pill boxes, text reminders) [31]–[33]. Successful interventions to improve retention in HIV care have required more complex and intensive efforts to decrease unmet needs, decrease structural barriers and reduce substance abuse [34]. Given the suboptimal state of retention in HIV care and adherence to HAART, it is critical to identify additional modifiable drivers to inform evidence-based interventions to optimize HIV care.

Patient satisfaction represents an innovative focus for retention and adherence intervention efforts. Its innovation derives from applying the business model of customer satisfaction to improve patient adherence to care. Additionally, interventions to improve patient satisfaction with the overall care experience are not directly dependent on efforts to explicitly change patient behavior. Patient satisfaction reflects the patient's perception of the entire care process, and improving satisfaction metrics lies within the power of a clinic or institution.

Research indicates that provider and organizational factors play a large role in how patients evaluate their provider and overall clinic care [8], [35]. Several empirical studies have shown that training in patient-centered communication and audit feedback can help providers improve communication skills in ways that increase patient satisfaction [36], [37]. Furthermore, organizational factors like increasing the time allowed for provider visits and ensuring continuity of care with the same provider can increase patients' satisfaction with their provider and overall care [38]–[40]. Continued progress in studying patient satisfaction requires not only additional evaluation of its effects on health outcomes, but also developing an understanding of the particular mechanisms or processes through which beneficial results are achieved.

The development of successful interventions to improve retention in HIV care and adherence to HAART requires a better understanding of how patient satisfaction impacts those constructs. The exact mechanisms explaining the linkages between these constructs remain unclear. Additionally, it remains unclear which component or components of the care experience most strongly influence retention and adherence. Several studies, including a previous study based on this dataset, have reported that patients' evaluation of their provider correlates the strongest with their overall satisfaction [35], [41], [42]. However, the provider characteristic most predictive of overall patient satisfaction may differ from those that may affect clinical outcomes. For example, provider training in problem solving focused adherence counseling techniques, as proposed in Wilson et al. [43], may have more influence on adherence than providers' interpersonal and general communication skills. Future research directions need to include prospective quantitative studies to: 1) better understand which component or components of the care experience are most predictive of overall patient satisfaction, medication adherence and retention in HIV care, 2) quantify how the strength of association changes over time as care progresses and what factors significantly influence those trends, and 3) establish causal direction.

This study has several methodological strengths. Our practice-based model incorporates the business model of customer satisfaction with the clinical end point of HIV suppression. The study took place at 2 clinic sites. It primarily included a low-income minority population, which often has low rates of adherence to care and worse clinical outcomes [44]. This population would stand to gain the most from interventions to improve adherence to care.

This study has certain limitations. Although our study supports the proposed causal linkages between overall patient satisfaction, retention in HIV care, adherence to HAART, and HIV suppression, correlational data cannot provide definitive evidence of causality. Emerging consensus, however, suggests that such data, when examined through structural equation modeling, can help researchers articulate, clarify and evaluate causal explanations between constructs of interest [45]. Study eligibility required enrollment in clinic for at least one year and thus excluded patients new to HIV clinic. New clinic patients may have greater risk of being lost to follow-up. At the same time, new clinic patients have not formed any behavioral patterns of retention or adherence yet, may be more impressionable [46], and as a result, initial care experiences may have a greater effect on retention and adherence. At present, the relationship between satisfaction and adherence to HIV care in new clinic patients remains unclear. Furthermore, participants received HIV care at the VA and a public clinic, and study findings may not generalize to patients in other settings. Lastly, our model's explanatory power is limited to its included constructs. Our model should be extended in further research by including other predictors of retention in HIV care and adherence to HAART (e.g. patient attributes like adherence self-efficacy and outcome expectations, provider attributes like adherence problem solving counseling skills, etc). The extension of our model to include these and other variables may clarify patient satisfaction's relative contribution to retention and adherence.

### Conclusion

This study identified retention in HIV care and adherence to HAART as intervening constructs through which patient satisfaction influences HIV outcomes. Our data raises the intriguing possibility that interventions aimed at improving the patient care experience by improving contextual components of care (i.e. who, where and how care is provided) could affect outcomes without actually targeting objective clinical performance measures. Our findings suggest that patient satisfaction could serve as an innovative target for interventions to improve HIV outcomes.
